# Bleaching efficacy and enamel effects of tricalcium phosphate dentifrices containing charcoal or hydrogen peroxide

**DOI:** 10.1590/0103-644020256740

**Published:** 2026-04-10

**Authors:** Guilherme Silva dos Santos, Amanda Ferreira Felix, Iago César Ribeiro Teles Matos, Matheus Kury, Vanessa Cavalli

**Affiliations:** 1 Operative Dentistry Division, Department of Restorative Dentistry, State University of Campinas, Piracicaba Dental School, 901 Limeira Avenue, Piracicaba, São Paulo 13414-903, Brazil; 2 Dental Research Division, School of Dentistry, Paulista University, São Paulo, SP, Brazil. 1212

**Keywords:** Tooth Bleaching, Hydrogen Peroxide, Dentifrices, Charcoal

## Abstract

This study aimed to evaluate the bleaching efficacy and effects of dentifrices containing tricalcium phosphate (TCP) with activated charcoal (COAL) or hydrogen peroxide (HP) on dental enamel, compared with monofluorophosphate (MFP). Bovine enamel-dentin discs (n=10/groups) were stained and divided into TCP/HP, TCP/COAL, MFP/HP, MFP/COAL, and C (control - remineralizing solution) groups. Simulated toothbrushing (5.000 cycles) was performed, representing 6 months of clinical condition. The whiteness index (ΔWI^D^), color changes (ΔE_00_, ΔL, Δa, and Δb), and surface microhardness and roughness (SMH, Ra) were evaluated after staining and treatments. Morphology and mineral content were analyzed under scanning electron microscopy and energy-dispersive X-ray spectroscopy. Data were analyzed using ANOVA/Tukey or Bonferroni tests (α=5%). MFP/COAL demonstrated significantly higher Δa and Δb than C. A statistically significant difference was observed in the TCP groups only for Δa. MFP-containing groups exhibited significantly higher ΔE00 than C. All groups showed negative ΔWI^D^. MFP/COAL promoted greater darkening than TCP-containing and C groups (p<0.05). No statistical difference was detected among groups for ΔL, SMH, Ra, and %SHL (microhardness loss) (p>0.05). No morphological alterations were observed. TCP/HP showed lower mean Ca/P values. Therefore, dentifrices containing TCP associated with HP or COAL showed no bleaching efficacy and no harmful impact on enamel surface properties.

## Resumo

Este estudo avaliou a eficácia clareadora e os efeitos de dentifrícios contendo tricálcio fosfato (TCP) associado a carvão ativado (COAL) ou peróxido de hidrogênio (HP) sobre o esmalte dentário, comparados a dentifrícios contendo monofluorfosfato de sódio (MFP). Discos de esmalte-dentina bovino (n=10/grupo) foram pigmentados e divididos nos grupos: TCP/HP, TCP/COAL, MFP/HP, MFP/COAL e controle (C - solução remineralizante). Realizou-se escovação simulada (5.000 ciclos), representando aproximadamente seis meses de uso clínico. Foram avaliados o índice de brancura (ΔWI_D_), alteração de cor (ΔE_00_, ΔL, Δa e Δb), microdureza de superfície (SMH, %SHL) e rugosidade (Ra), antes e após os tratamentos. A morfologia superficial e o conteúdo mineral foram analisados por microscopia eletrônica de varredura e espectroscopia por dispersão de energia de raios X. Os dados foram submetidos à ANOVA e testes de Tukey/Bonferroni (α=5%). O grupo MFP/COAL apresentou Δa e Δb significativamente maiores em relação ao controle, enquanto os grupos TCP mostraram diferença estatística apenas para Δa. Os dentifrícios com MFP exibiram valores de ΔE_00_ significativamente maiores que o grupo controle. Todos os grupos apresentaram ΔWI_D_ negativo, sendo que MFP/COAL promoveu escurecimento mais acentuado do que os grupos TCP e controle (p<0,05). Não foram observadas diferenças significativas entre os grupos para ΔL, SMH, Ra e %SHL (p>0,05), nem alterações morfológicas na superfície do esmalte. O grupo TCP/HP apresentou valores médios de Ca/P mais baixos. Conclui-se que os dentifrícios contendo TCP associados a HP ou COAL não demonstraram eficácia clareadora significativa, e não causaram efeitos prejudiciais às propriedades da superfície do esmalte.

**Figure:**
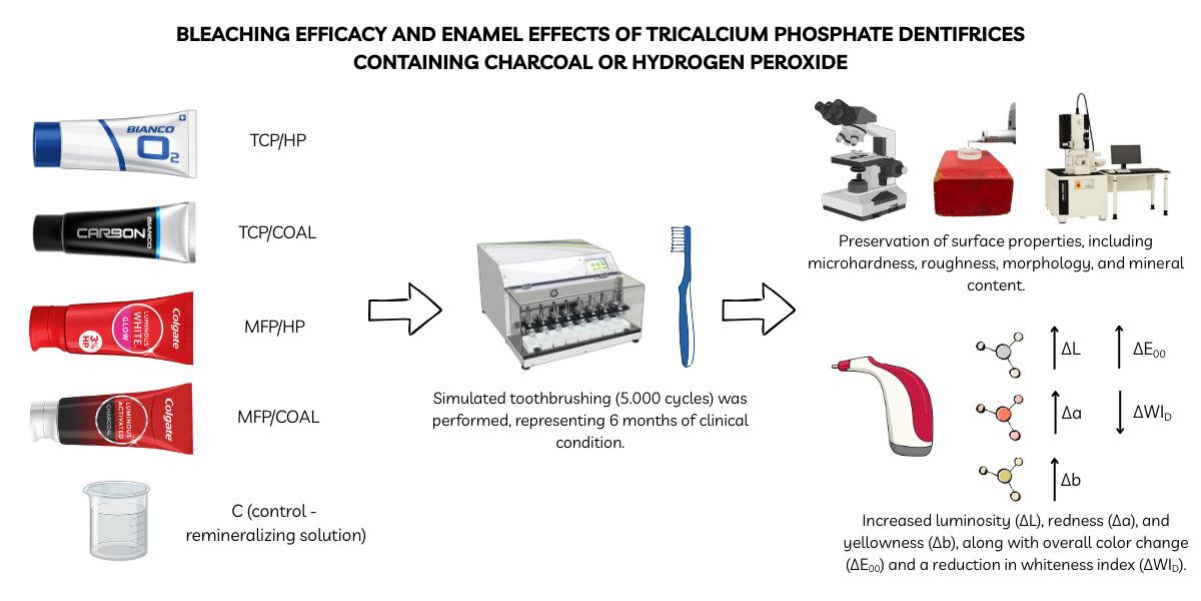

## Introduction

The pursuit of a white smile extends beyond mere aesthetics; it profoundly influences self-esteem, psychological behavior, and social interactions. For this reason, dental bleaching has become a hugely popular cosmetic procedure among patients^1^. This minimally invasive process uses gels containing hydrogen peroxide (HP) or carbamide peroxide (CP) at varying concentrations. The mechanism of action of these peroxides involves breaking down the HP into reactive oxygen species, which, due to their low molecular weight, interact with dentin pigments (chromophores) responsible for the color of dental structures^2^.

Dental color is also influenced by externally derived pigments from drinks such as tea, coffee, and red wine, as well as from smoking habits^3^. Consequently, various bleaching dentifrice formulations have been introduced to the market in recent years to mitigate the effects of these pigments^4^. Common strategies for achieving a bleaching effect include increasing the concentration of abrasives or incorporating harder abrasives, but these approaches raise concerns regarding increased enamel roughness^5^ and decreased microhardness^6^. However, the impact of such strategies on enamel properties remains a topic of debate, as there is no consensus on whether these changes affect the enamel integrity^7^. Furthermore, many of these dentifrice formulations contain agents with remineralization potential such as sodium fluoride (NaF), stannous fluoride (SnF_2_), amine fluoride (AmF), tricalcium phosphate (TCP), and monofluorophosphate (MFP).

Recently, a dentifrice containing monofluorophosphate (MFP) combined with the abrasive activated charcoal (COAL) demonstrated the ability to induce color change *in vitro* ^8^. According to Palandi et al. (2020)^9^, products containing activated charcoal altered tooth color, although this change was significantly inferior to that with a 16% CP bleaching gel. Furthermore, the abrasiveness of activated charcoal has been shown to affect enamel surface roughness^10^ negatively, microhardness^11^, and surface morphology^9^, depending on the duration of exposure. Moreover, activated charcoal has been incorporated into dentifrices containing tricalcium phosphate (TCP), demonstrating its ability to remove extrinsic enamel pigmentation partially. However, its effects on surface properties were not evaluated^12^. TCP functions by preventing premature interactions between fluoride and calcium, resulting in a low-dose fluoride release system that enhances fluoride’s efficacy in reducing dental surface property loss^13^. A previous study demonstrated that dentifrices containing TCP, without a bleaching purpose, increased enamel surface microhardness^14^.

Another alternative dentifrice claiming to bleach teeth is based on low hydrogen peroxide (HP) concentrations^15^. These products promise to chemically modify the pigments that intrinsically adhere to the dental, reducing their intensity and the appearance of discoloration^16^. Nevertheless, the low concentration of HP, combined with its inherent instability, can directly compromise its bleaching action^17^. Furthermore, dentifrices containing HP, when combined with a remineralizing agent, may stabilize enamel surface properties, reducing negative alterations^18^. Despite a study analyzing the efficacy of dentifrices containing TCP associated with COAL^12^, no reports in the literature assess the efficacy and effects of dentifrices containing TCP associated with 3% HP on color change, particularly about the attempt to remove intrinsically adherent pigments through the action of HP, and its influence on enamel surface properties.

Therefore, due to the various compositions of bleaching dentifrices currently available, this study aimed to evaluate the combination of "bleaching” agents such as activated charcoal (COAL) or hydrogen peroxide (HP) with TCP, compared to a standard agent with remineralization potential (MFP), regarding colorimetric changes and enamel surface properties. The null hypotheses tested were that dentifrices containing TCP would not affect: i) color change, ii) surface microhardness, and iii) surface roughness after simulated brushing.

## Materials and methods

### Experimental design

Fifty bovine enamel-dentin discs were randomly distributed into treatments with different dentifrices (n = 10/group): TCP/HP; TCP/COAL; MFP/HP; MFP/COAL; C (control). Enamel surface microhardness (SMH), percentage of surface microhardness loss (%SHL), surface roughness (Ra e ΔRa), color parameters (ΔL, Δa, Δb), color change (ΔE_00_), and whiteness index (ΔWI_D_) were evaluated before (T_0_) and after brushing (T_1_). Surface morphology (SEM) and energy-dispersive X-ray spectroscopy (EDS) were evaluated after the treatments. 

### Specimens’ preparation

Bovine incisors were sectioned using a bench drill (FSB16, Pratika, Schulz, SP, Brazil) to obtain enamel-dentin discs measuring 5 × 3 mm, maintaining both tissues in the same specimen. Initially, the discs were mounted on stubs with the dentin side facing upward and fixed with wax, allowing the dentin to be flattened from the pulpal side to obtain parallel enamel and dentin surfaces. The discs were then removed, repositioned with the enamel side facing upward, and polished using a polishing machine (Arotec, Cotia, São Paulo, Brazil) with #600- and #1200-grit silicon carbide sandpapers (Norton Saint-Gobain, Guarulhos, SP, Brazil), followed by polishing with 1 µm diamond paste to obtain a flat, smooth surface suitable for microhardness and roughness measurements. The polished discs were ultrasonically cleaned in distilled water for 10 min between sandpaper use and after final polishing to remove residual particles. Samples exhibiting white spots, cracks, or other defects were excluded. The lateral dentin surfaces of the specimens were protected with a colorless varnish. Subsequently, the specimens were mounted on acrylic plates using sticky wax, fully covering the exposed dentin surfaces and leaving only the enamel surface exposed.

### Artificial enamel pigmentation

The enamel surface was pigmented using a buffered black tea solution (pH = 7.0) (Camellia Sinensis), following the modified method of Sulieman et al. (2003)^19^. Black tea (2 g) was diluted in 100 mL of distilled water for 5 min. After filtration, the specimens were immersed in the solution for 24 h with agitation at room temperature. Subsequently, the pigmented specimens were cleaned with pumice and a Robinson brush to remove non-adhered particles. They were then stored in remineralizing solution (1.5 mM Ca; 0.9 mM PO_4_; 150 mM KCl in a 20 mM Tris buffer solution, pH 7.0), following the protocol based on Viana et al. (2021)^20^, for 7 days (with solution changes every 2 days) to stabilize the color before starting the treatments, in an incubator at 37ºC. After stabilization, an initial color analysis was performed, and using the L* coordinate, the specimens were randomized into five experimental groups, as described previously. 

### Experimental groups

The specimens were divided into groups (n = 10/group):

- TCP/HP: Brushing with dentifrice containing Tricalcium Phosphate and Hydrogen Peroxide (Bianco Dental O_2_);
- TCP/COAL: Brushing with dentifrice containing Tricalcium Phosphate and Activated Charcoal (Bianco Dental Carbon);
- MFP/HP: Brushing with dentifrice containing Sodium Monofluorophosphate and Hydrogen Peroxide (Colgate Luminous White Glow);
- MFP/COAL: Brushing with dentifrice containing Sodium Monofluorophosphate and Activated Charcoal (Colgate Luminous White Activated Charcoal);
- C: Control (brushing with remineralizing solution).

Table 1 represents the composition and pH of the dentifrice formulations.

### Solution’s preparation and pH analyses

The remineralizing solution was prepared containing 1.5 mM Ca, 0.9 mM PO4, 150 mM KCl, and 20 mM Tris, pH 7.0 (Queiroz et al., 2008). The slurries of each dentifrice were diluted in distilled water at a 1:3 ratio (dentifrice, g / distilled water, mL), as previously described for the groups.

The pH of the tested dentifrice slurries was measured in triplicate using a pHmeter (Equilam, Diadema, SP, Brazil) coupled with a potentiometer (Orion Research Incorporated, Boston, MA), calibrated with pH 4.0 and 7.0 standards (Viana et al., 2021).

**Table 1:** Composition of the dentifrices used.

Dentifrice	Manufacturer	Composition	Agents	pH
Bianco Dental O_2_	Lima & Pergher Comércio S/A	Water, glycerin, hydrated silica, sodium lauryl sulfate, flavor, PVP, honey, cocamidopropyl betaine, xanthan gum, polyglyceryl-3 caprylate, sodium benzoate, citric acid, carrageenan, sodium saccharin, tocopheryl acetate, panthenol, CI 74160	Active ingredient: Tricalcium Phosphate “Bleaching” ingredient: Hydrogen Peroxide	5.93 ± 0.06
Bianco Dental Carbon	Lima & Pergher Comércio S/A	Water, glycerin, hydrated silica, sodium lauryl sulfate, xylitol, flavor, maltodextrin, cellulose gum, sodium benzoate, zinc lactate, sucralose, citrus limon (lemon) fruit extract, citrus auratium dulcis (orange) fruit extract, zingiber officinale extract.	Active ingredient: Tricalcium Phosphate “Bleaching” ingredient: Activated Charcoal Powder	7.51 ± 0.10
Colgate Luminous White Glow	Colgate Palmolive Company, São Paulo, SP, Brazil	Propylene glycol, dicalcium pyrophosphate, PEG/PPG-116/66 copolymer/poloxalene, aroma, sodium lauryl sulfate, tetrasodium pyrophosphate, sodium, disodium pyrophosphate, silica, sucralose, BHT, eugenol, linalool.	Active ingredient: Sodium Monofluorophosphate “Bleaching” ingredient: Hydrogen Peroxide	6.97 ± 0.02
Colgate Luminous White Activated Charcoal	Colgate Palmolive Company, São Paulo, SP, Brazil	Water, hydrated silica, sorbitol, calcium pyrophosphate, glycerin, PEG-12, Pentasodium triphosphate, tetrapotassium pyrophosphate, aroma, sodium lauryl sulfate, cellulose gum, sodium saccharin, xanthan gum, cocamidopropyl betaine, CI 16035, CI 42090, CI 19140, limonene.	Active ingredient: Sodium Monofluorophosphate “Bleaching” ingredient: Activated Charcoal Powder	7.41 ± 0.02

### Brushing protocol

Mechanical brushing was performed to simulate 6 months of brushing, equivalent to 5,000 cycles, following the methodology outlined by Palandi et al. (2020)^9^. All specimens were brushed with a soft-bristle brush (Oral-B Clean Indicator) utilizing a mechanical brushing machine (MSet, Nucci ME, São Carlos, SP, Brazil). This equipment facilitated the simultaneous brushing of 10 specimens per cycle, which were immersed in a remineralizing solution (control group) or a tested dentifrice solution diluted in distilled water (1:3 v/v). The brushing cycles occurred continuously once a day at 5 Hz under a load of 200 g, replicating the force typically applied during oral hygiene routines. Following brushing, the specimens were rinsed in running water, gently dried with soft paper, and subsequently immersed in a remineralizing solution for 24 h to facilitate subsequent analyses. 

### Colorimetric evaluation

Three measurements were performed on the enamel surface using a digital spectrophotometer (EasyShade, Vita Zahnfabrik, Bad Säckingen, Germany), positioned against a white background inside a standardized light booth (GTI Mini Matcher MM 1e, GTI Graphic Technology Inc., Newburgh, NY, USA) using the "daylight" option to standardize the readings. The device's measuring tip was positioned perpendicular to the enamel surface to ensure consistent, accurate color readings. Color evaluations were conducted post-pigmentation (T_0_) and 24 h after dentifrice treatments (T_1_) to assess color parameters L* (black to white), a* (green to red), and b* (blue to yellow), as well as h (hue) and C (chroma). Color change was evaluated employing the CIEDE2000 formula (ΔE_00_) = [(ΔL´/KLSL)^2^ + (ΔC´/KCSC)^2^ + (ΔH´/KHSH)^2^ + RT*(ΔC´/KCSC)(ΔH´/KHSH)]^1/2^. The whiteness index for dentistry (WI_D_) and the difference in whiteness index (ΔWI_D_) were computed using the equations: WI_D_ = 0.511L - 2.324a* - 1.100b* and ΔWI_D_ = final WI_D_ - initial WI_D_. The perceptibility (PT) and acceptability (AT) thresholds for ΔE_00_ were 0.8 and 1.8, respectively^21^, while for WI_D_ were 0.72 (PT) and 2.62 (AT)^22^.

### Surface microhardness (SMH)

The initial surface microhardness (SMH) was assessed by conducting three indentations in the central area of each specimen using a Knoop-type hardness tester (Future Tech-FM-1e, Tokyo, Japan). A static load of 50 g was applied for 5 seconds, with a 100 µm spacing between indentations. Analyses were conducted post-pigmentation (T_0_), with the average SMH values of all specimens recorded at 353.9 kg/cm^2^. A 10 % variation (+/-) from the mean values was employed for sample selection. Following this, specimens were randomly assigned to five experimental groups, with initial microhardness values showing no statistical differences among them (ANOVA; p > 0.05). Therefore, microhardness was assessed 24 h after the brushing periods (T_1_). At both T_0_ and T_1_, the percentage of surface microhardness loss [%SHL = Initial SMH - Final SMH / Initial SMH * 100] was calculated.

### Surface roughness (Ra)

The average surface roughness (Ra, µm) was assessed using a roughness meter (Surfcorder SE 1700, Kosalab) with a cut-off of 0.8 mm at the initial stage (T_0_) and post-treatments (T_1_). Specimens were individually affixed to an acrylic base and aligned parallel to the equipment's surface. The measuring tip of the equipment was positioned perpendicular to the surface, and three measurements were taken per sample, rotating the specimen 45º between each measurement. The average Ra value for each sample was then calculated. The average change in surface roughness (ΔRa) was determined by subtracting the T_0_ value from the T_1_ value.

### Scanning electron microscopy (SEM) and energy-dispersive X-ray spectroscopy (EDS)

Five representative specimens from each group were selected and analyzed for their morphology (SEM) and mineral content (EDS) using scanning electron microscopy (SEM - JEOL - JSM, 6460 LV, Tokyo, Japan) and energy-dispersive X-ray spectroscopy software (EDS, Vantage System - Easymicro Noran Instruments, Middleton, Wisconsin, USA). After the treatments, the specimens were cleaned in an ultrasonic bath (Ultra Cleaner, Unique, Indaiatuba, SP, Brazil) for 10 min and dried for 24 h in an oven at 37ºC. Once dried, the specimens were sputter-coated with a tin-carbon layer and analyzed by automated image analysis using SEM at 15 kV in vacuum mode (45 Pa)^23^. Images were acquired at 1000x magnification. Concurrently with the SEM image acquisition, the EDS software provided semi-quantitative data on the percentage of chemical elements (atomic percentage) present in the selected area of the sample surface.

### Statistical analyses

The collected data were submitted to exploratory analyses for normality and homoscedasticity using the Shapiro-Wilk and Levene tests (p > 0.05), respectively, in GraphPad Prism, version 10 (10.2.3). The results of ΔL, Δa, Δb, ΔE_00_, %SHL, ΔRa, and ΔWI_D_ were submitted to one-way ANOVA and Tukey post hoc tests. The SMH and Ra values assessed over time were submitted to a one-way repeated-measures ANOVA, followed by a Bonferroni post hoc test. Additionally, the Dunnett test was conducted to compare the control group (brushing with remineralizing solution) with the experimental groups. A significance level of 5% was set for all analyses.

## Results

### Evaluation of Euclidean coordinates (L*, a* e b*)

**Figure 1:**
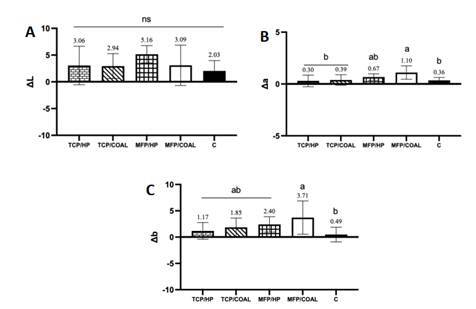
(A): Graphical representation of mean values and standard deviation of ΔL. Bars connected by a horizontal line and "ns" indicate that they are not significantly different from each other; Figure 1 (B): Graphical representation of mean values and standard deviation of Δa. Numbers show the means of each group. Different letters indicate significant differences among groups; Figure 1 (C): Graphical representation of mean values and standard deviation of Δb. Numbers show the means of each group. Different letters indicate significant differences among groups.

The mean and standard deviation of the color evaluation data for L* (black-white), a* (green-red), and b* (blue-yellow) are shown in Figure 1. In terms of ΔL (Figure 1A), no significant differences were observed among the groups (p > 0.05). However, the TCP/HP and TCP/COAL groups exhibited significantly lower Δa values (p < 0.05) than the MFP/COAL group (Figure 1B), which was the only group with values higher than the control. Regarding Δb, MFP/COAL exhibited significantly higher values compared to the control (C) (p < 0.05), while no significant differences were observed among the dentifrice groups (p > 0.05).

### Color change (ΔE_00_) and Whiteness Index (ΔWI_D_)

**Figure 2:**
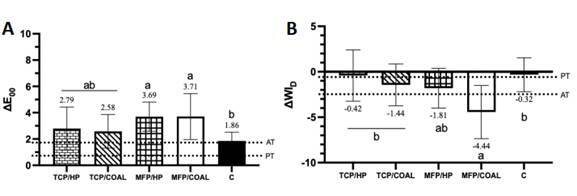
(A): Graphical representation of mean values and standard deviation of ΔE_00_. Numbers show the means of each group. Different letters indicate significant differences among groups. Horizontal lines represent the perceptibility (PT) and acceptability (AT) thresholds, set at 0.8 and 1.8, respectively; Figure 2 (B): Graphical representation of mean values and standard deviation of ΔWI_D_. Numbers show the means of each group. Different letters indicate significant differences among groups. Horizontal lines represent the perceptibility (PT) and acceptability (AT) thresholds, set at 0.72 and 2.62, respectively.

All groups achieved mean ΔE_00_ values above the 50:50% perceptibility (PT) and acceptability (AT) thresholds (Figure 2A), but only the MFP-containing groups showed statistically significant differences compared to the control group (p > 0.05). All groups presented negative ΔWI_D_ results (Figure 2B). The TCP/HP and C groups showed mean ΔWI_D_ values below the perceptibility threshold (0.8). Moreover, the MFP/COAL dentifrice not only demonstrated significantly more negative results compared to the groups containing TCP, but was also the only one to show a statistically significant difference from the control.

### Surface microhardness (SMH) and Surface roughness (Ra)

Table 2 shows that no significant differences in %SHL were observed among the groups (p > 0.05). Additionally, no significant changes in mean SMH values were detected over time (p > 0.05). Surface roughness remained unaffected by brushing, as no statistical differences were found among the groups or across time points (p > 0.05).

**Table 2:** Mean and standard deviation values of surface microhardness (SMH) and surface roughness (Ra) for each group and over time, and percentage of surface hardness loss (%SHL) and surface roughness change (ΔRa) between T_1_ and T_0._

	Surface Microhardness (SMH)			Surface Roughness (Ra)		
	T_0_	T_1_	%SHL	T_0_	T_1_	ΔRa
TCP/HP	356,4 (29,5)	347,7 (30,8)	1,8%	0.055 (0.009)	0.055 (0.006)	0
TCP/COAL	353,8 (27)	351,8 (27,1)	0,3%	0.06 (0.021)	0.054 (0.012)	-0.006
MFP/HP	349,5 (24,9)	344,8 (36,6)	1,2%	0.064 (0.039)	0.064 (0.017)	0
MFP/COAL	351,5 (25,1)	352,3 (21)	-0,3%	0.062 (0.012)	0.057 (0.013)	-0,005
C (control)	358,1 (28,6)	358,8 (37,1)	-0,7%	0.063 (0.022)	0.059 (0.019)	-0.004

Absence of letters denotes no statistically significant differences among groups or time points

### Scanning electron microscopy (SEM) and energy-dispersive X-ray spectroscopy (EDS)

**Figure 3:**
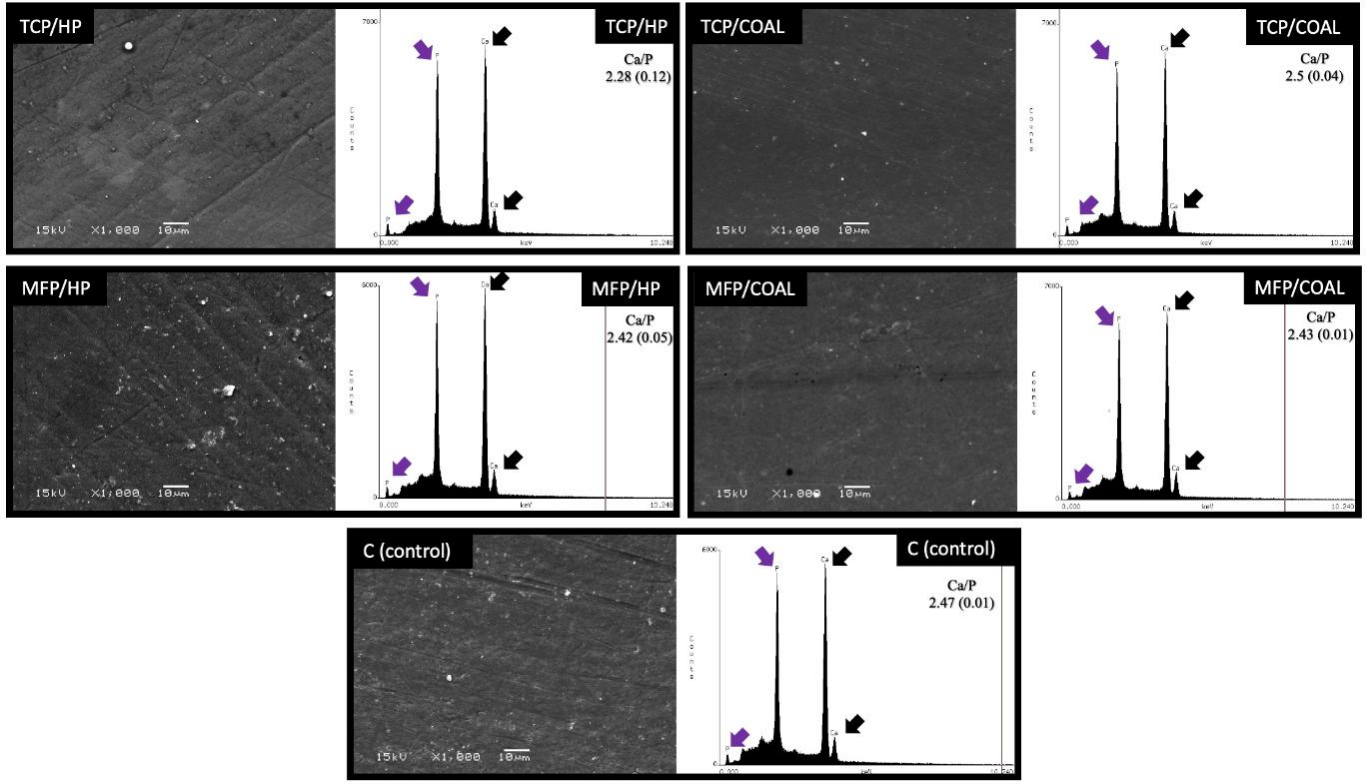
Representative SEM images indicate that there were no changes in the enamel surface, with no observable porosities or irregularities. Despite visible marks resulting from simulated brushing, all groups exhibited similar behaviors. In the EDS plots, black arrows denote Ca (calcium) peaks, while purple arrows indicate P (phosphorus) peaks. Additionally, the TCP/HP group had the lowest mean Ca/P ratio among all groups.

Representative SEM images (Figure 3) show no observable surface changes in any of the groups, despite treatment with abrasive agents, such as activated charcoal, or chemicals, such as hydrogen peroxide. The enamel surfaces remained largely unaffected following the treatments, showing no signs of porosity, irregularities, or demineralized areas, and only revealing brushing marks, similar to those observed in group C (control). Semi-quantitative EDS analysis indicated that the TCP/COAL exhibited the highest mean Ca/P ratio. In contrast, MFP/COAL and MFP/HP exhibited intermediate values, while TCP/HP showed lower mean Ca/P values following brushing. 

## Discussion

In this study, the effects of simulated brushing on the dental enamel surface using dentifrices containing tricalcium phosphate (TCP) combined with activated charcoal (COAL) or 3% hydrogen peroxide (HP) were thoroughly evaluated. The study hypothesized that the presence of TCP in the dentifrices would not affect color change, surface microhardness, or surface roughness. As a result, the first null hypothesis was accepted, as brushing with the dentifrices over a simulated 6-month period did not result in a significant color change (ΔE_00_) compared to the control group (without dentifrice). The absence of a significant overall color change may be attributed to the low concentration and dilution of bleaching agents in dentifrices, which are insufficient to influence enamel intrinsic color. Moreover, all tested dentifrices increased dental luminosity (ΔL), consistent with previous literature^24^.

Unexpectedly, all groups showed positive values for both Δa and Δb, indicating a trend toward increased tooth saturation after brushing with dentifrices. The significantly higher Δa values observed for the MFP/COAL compared to both C and TCP-containing groups, and the higher Δb values in comparison to the control group, suggest that the dentifrice combining a conventional remineralization agent (MFP) with activated charcoal presented greater difficulty in removing reddish and yellowish pigments, which are typically deposited by black tea. Previous studies evaluating the efficacy of bleaching dentifrices after brushing and staining protocols have reported similar outcomes, in which brushing with Colgate Luminous White Activated Charcoal containing MFP and COAL also resulted in higher mean values on the a* and b* axis, confirming our findings^5^ ^,^ ^8^. Therefore, the difficulty in removing reddish pigments appears to be linked to the intrinsic composition of this dentifrice. 

Although activated charcoal presents high porosity and extensive surface area that enable adsorption of superficial pigments, reddish and yellowish stains^25^, exhibit complex molecular structures and strong affinity for the enamel matrix^25^ ^,^ ^12^. As a result, their removal by simple surface adsorption is limited. Previous studies indicate that even after brushing with charcoal-based dentifrices, stains of this type are only partially removed, since pigments penetrate deeper into the enamel, beyond the effective action of charcoal particles^8^ ^,^ ^12^.

For color analysis, a prophylaxis was performed prior to the application of the dentifrices to remove loosely adhered particles from the enamel surface^26^. This procedure was intended to evaluate the effects of the "bleaching" agents by allowing them to interact with intrinsically bound pigments. This step was particularly relevant for dentifrices containing hydrogen peroxide (HP), given their expected mechanism of action, which involves oxidation of chromophores. Regarding color change, dentifrices containing TCP, whether combined with HP or COAL, showed no significant differences compared to the control group. In contrast, MFP-containing groups showed a significant increase in ΔE_00_ values compared with the control. These results suggest that TCP-based formulations promote greater optical stability of the enamel surface, possibly by limiting pigment adsorption. While ΔE_00_ provides valuable information about overall tooth color change, it offers limited insight into the specific efficacy of a bleaching agent^27^. 

For this reason, the whiteness index for dentistry (ΔWI_D_) was also evaluated, with higher values indicating whiter samples and more negative values indicating darker samples^27^. Despite simulated brushing increasing luminosity (L*), there was a general trend towards darkening, driven by increases in redness (a*) and yellowness (b*). This result directly impacted the ΔWI_D_ values, as all groups exhibited negative values by the end of the experiment. In this context, it is important to highlight that no bleaching effect was observed in any of the groups. The inclusion of dentifrices containing COAL or HP, along with MFP, aimed to compare the practical outcomes of these agents. 

Interestingly, the MFP/COAL group demonstrated the most pronounced darkening effect (the most negative ΔWI_D_) compared with both the TCP-containing and control groups. This outcome may be attributed to the specific characteristics of activated charcoal in this formulation, such as its dark color and irregular morphology. Although activated charcoal is known to adsorb extrinsic pigments^5^, it is plausible that, in this context, the dark color of the slurry may have facilitated the deposition of dark pigments rather than removal. Differences in dentifrices' abrasiveness may also have influenced the observed WI_D_ values. Although RDA measurements were not performed and manufacturers generally do not disclose this information, TCP-based formulations may have shown slightly higher abrasiveness than MFP, which could have contributed to the observed color outcomes. Conversely, the MFP/COAL dentifrice, characterized by its darker slurry and likely lower abrasiveness, exhibited the most significant reduction in whiteness. This interpretation remains speculative and should be verified in future studies that concurrently evaluate RDA values and their influence on optical parameters. This speculation can be supported by findings from a study conducted by Ribeiro et al. (2024)^28^, who also identified that dentifrices containing activated charcoal tend to result in darkening compared to other bleaching agents.

Although activated charcoal is marketed as a natural bleaching product^29^, a previous study found that this abrasive component is less effective than a peroxide-based agent in altering dental color^9^. This is due to several factors that determine its efficacy, such as charcoal’s particle size, shape, hardness, concentration, and distribution^29^. However, manufacturers typically do not provide this information, making it difficult to compare groups that use charcoal as a bleaching agent adequately. Furthermore, the presence of chemical components intended to promote color changes, such as hydrogen peroxide (HP) in dentifrice compositions, also failed to produce significant results^8^. The low peroxide concentration in dentifrices directly influences their efficacy, and dilution with distilled water, as well as limited contact time during brushing, can negatively affect their bleaching action^30^.

Tricalcium phosphate (TCP) in dentifrices promotes remineralization and enamel strengthening by providing a low-dose fluoride release system^13^. Although our results showed that tricalcium phosphate did not produce significant changes in enamel surface properties, including surface microhardness (SMH) and surface roughness (Ra), its use did not adversely affect these parameters, consistent with a previous study^18^. This suggests that although this agent with remineralizing potential may play an important role in oral health^31^, its presence in the tested dentifrice did not result in a significant difference compared to the control group. Furthermore, despite the different formulations of the tested dentifrices, all groups maintained consistency in enamel surface microhardness and roughness after simulated brushing, leading the authors to accept the second and third null hypotheses.

Therefore, the absence of statistical difference with the control group regarding enamel surface properties, such as microhardness and roughness, may be attributed to the lack of a remineralization process. This outcome is primarily explained by the use of sound enamel samples, which had not undergone prior demineralization, a necessary condition for the release of remineralizing agents^32^. Given that fluoride in the form of monofluorophosphate (MFP) primarily functions to reduce demineralization and enhance remineralization, similar to TCP, as previously mentioned, its effect may have been limited under the conditions of this study, such as the fact that MFP becomes bioavailable upon hydrolysis by phosphatase enzymes present in human saliva^33^. However, as this study used a remineralizing solution to simulate the oral environment and employed sound enamel, the MFP's remineralizing effect was consequently limited. It may not have been detected under the in vitro conditions tested.

In a way, the presence of abrasives is essential for the efficacy of dentifrices in removing only extrinsic pigmentation. However, as mentioned before, the addition of activated charcoal to dentifrices can have adverse effects on the surface of dental enamel, such as increased surface roughness^9^ and decreased surface microhardness^6^. A study conducted by da Silva et al. (2024)^5^ demonstrated that a dentifrice containing activated charcoal showed significantly higher wear values than a conventional dentifrice after 100,000 brushing cycles (approximately 10 years). On the other hand, the differences observed in the present study may be attributed to variations in the brushing protocol. While the present investigation simulated only 5.000 brushing cycles, compared to 100.000 reported by da Silva et al. (2024)^5^, these milder conditions likely minimized mechanical abrasion, which may explain the absence of enamel damage in the charcoal-containing groups. 

An alternative to replace charcoal and enhance the bleaching effect was the addition of hydrogen peroxide (HP) to the dentifrice composition. However, this agent can also alter the surface properties of dental enamel^30^. Notably, the HP-containing groups showed higher average microhardness loss than the other groups. This observation suggests that HP may have a more pronounced effect on surface properties, possibly due to its bleaching and oxidizing properties, which may reduce the pH of dentifrices^18^.

Among the bleaching products tested, the dentifrices containing HP had the lowest pH values compared to those containing COAL (Table 1). Despite all products exhibiting pH values above the critical threshold for enamel demineralization (5.5)^34^, only TCP/HP (pH = 5.93) approached this threshold. The products' acidity can be attributed to several factors. Firstly, the chemical instability of other ingredients and additives in combination with hydrogen peroxide can contribute to the lower pH^35^. Maintaining proper pH control is crucial to ensure the product’s safety for oral use and to prevent potential damage to dental or oral mucosa^35^. Additionally, the use of acidic bleaching products, such as TCP/HP, can alter the structure and mechanical properties of the tooth surface through enamel demineralization.

The use of hydrogen peroxide alone can cause adverse effects on dental enamel, including the loss of calcium and phosphate minerals^36^, leading to physical and chemical changes. Consequently, EDS analysis showed a semiquantitative decrease in Ca/P ratios in the TCP/HP group. Although both TCP/HP and TCP/COAL contain tricalcium phosphate that acts on the hydroxyapatite structure, the presence of hydrogen peroxide likely influenced this result due to the action of free radicals produced by the oxidation of organic and inorganic elements in the dental structure^36^. These findings must be carefully evaluated, as EDS analysis is relative (%), performed on a random section of the enamel. Thus, EDS analysis is a semi-quantitative method that should be used in conjunction with other methodologies to quantify enamel chemical composition after bleaching. 

Although the literature presents studies indicating that activated charcoal^9^ ^,^ ^37^ ^)^ and hydrogen peroxide^30^ promote topographic changes in dental enamel due to their abrasive potential and the action of free radicals resulting from the oxidation of organic and inorganic elements in dental, respectively, the SEM images (Figure 3) did not show noticeable surface changes in any of the groups. This lack of observable changes and the similarity to the control group (brushed with remineralizing solution) can be attributed to the presence of a remineralizing agent, whether TCP^14^ or MFP^36^, which were essential to obtain these results regarding the surface properties of enamel.

The limitation of this study is that it is an in vitro study, in which brushing was followed by prolonged pigmentation, unlike clinical practice, where pigmentation deposition is interspersed with brushing. Additionally, diluting the dentifrice with distilled water and limiting its contact with the dental substrate hinder the action of hydrogen peroxide. Furthermore, factors such as human saliva, salivary flow, acquired pellicle formation, and exposure to acidic agents were not simulated due to the in vitro nature of the experiment. It should also be noted that the exposure time and pigmentation duration used in this study were intentionally extended compared with those typically encountered in clinical practice.

## Conclusion

Brushing simulating 6 months, using dentifrices containing tricalcium phosphate associated with hydrogen peroxide or activated charcoal, did not show a bleaching effect nor interfere with enamel surface properties.

## Data Availability

The research data are available upon request.
